# Heat treatments of ginger root modify but not diminish its antioxidant activity as measured with multiple free radical scavenging (MULTIS) method

**DOI:** 10.3164/jcbn.18-41

**Published:** 2018-12-05

**Authors:** Yoshimi Sueishi, Hiroaki Masamoto, Yashige Kotake

**Affiliations:** 1 Department of Chemistry, Faculty of Science, Okayama University, 3-1-1 Tsushima-naka, Kita-ku, Okayama 700-8530, Japan; 2 RRINC-USA, Merced, CA 95340, USA

**Keywords:** ginger, antioxidant capacity, multiple free-radical scavenging method, MULTIS, ESR spin trapping

## Abstract

Ginger (*Zingiber officinale* Rosc.) root (or rhizome) has been reported to have antioxidant properties such as reactive oxygen species scavenging activities. Using multiple free-radical scavenging method, we have newly determined the scavenging abilities of ginger roots against five reactive oxygen species, i.e., HO^•^, O_2_^−•^, RO^•^, *tert*-BuOO^•^, and ^1^O_2_. After heating grated ginger roots at 80°C for 2 h, nearly 50% decrease in scavenging ability was recorded against ^1^O_2_ and *tert*-BuOO^•^. Conversely, the O_2_^−•^ scavenging ability increased by about 56% after heat treatment. Based on the antioxidant activity measurement of the ginger’s components, i.e., 6-gingerol, 6-shogaol, and zingerone, active species acting as antioxidant capacity of ginger was shown. Additionally, ginger’s antioxidant capacity was quantitatively compared with that of rosemary extract, indicating that rosemary is peroxyl specific scavenger while ginger has higher scavenging ability against HO^•^ and ^1^O_2_.

## Introduction

The rhizome (root) of ginger (*Zingiber officinale* Rosc.) is widely used as a spice and herbal medicine. The main pungent component gingerol has been shown to have a variety of biological properties, including anticancer, antioxidant, antiinflammation, and antifungal.^(1,2)^ Nevertheless, the effective component that is responsible to such activities is not well understood.

6-Gingerol, a ginger’s major pharmacologically active component is considered an important natural antioxidant of food origin and has been shown to decrease peroxidation of phospholipid liposomes *in vitro*.^(3,4)^ The other component 6-shogaol has many biological effects such as antibacterial and antioxidant activities. Zingerone and shogaol, the dehydrated form of gingerol, are formed during thermal processing and long-term storage of ginger roots.^(5)^ Based on the scavenging ability against a synthetic free radical compound, Dugasani *et al.*^(6)^ have demonstrated that 6-shogaol may have potent free radical scavenging properties. However, the direct scavenging abilities against various biologically-relevant active species have not been evaluated yet.

In previous studies, the scavenging activities of antioxidants are determined by using stable synthetic free radical such as DPPH (diphenyl picryl hydrazyl) or galvinoxyl.^(7)^ However, it is reasonable to speculate that the reactivity of antioxidants with stable synthetic radical may be drastically different from those with various biologically active species. Recently, ESR (electron spin resonance) spin-trapping based multiple free-radical scavenging (MULTIS) method has been developed.^(8,9)^ In that method, various reactive oxygen species were photochemically generated, against which antioxidant’s scavenging ability was quantified. Thus, direct scavenging abilities of hydrophilic antioxidants were determined in cattle and human sera against reactive oxygen species, such as hydroxyl, superoxide, and singlet oxygen.^(8,9)^ Also, the MULTIS method has been shown to be a useful method in the determination of comparative antioxidant capacity in plant specimens.^(10,11)^

In this study, both lipophilic and hydrophilic antioxidants in ginger were solubilized and diluted in a mixture of acetonitrile and water. Acetonitrile itself has negligible active species scavenging ability and was expected to act as a pure medium. After such modification, we have determined the direct scavenging abilities of gingers (*Zingiber officinale*) produced in Japan against five reactive oxygen species (hydroxyl radical, superoxide, alkoxyl radical, peroxyl radical, and singlet oxygen). This paper is the first to show that the MULTIS method is useful in the determination of the antioxidant activity in food. We found that the heat treatment of the ginger specimen either diminished or enhanced antioxidant activities, depending on the reactive oxygen species scavenged. Also, we elucidated the cause of heat-mediated alteration of the antioxidant capacity.

## Materials and Methods

### Materials

Spin trap 5,5-dimethyl-1-pyrroline *N*-oxide (DMPO) was purchased from Tokyo Chemical Ind. (Tokyo, Japan) and was used for the detection of hydroxyl (HO^•^) and alkoxyl (RO^•^) radicals. For superoxide (O_2_^−•^) and alkyl peroxyl radical (*tert*-BuOO^•^), 5-(2,2-dimethyl-1,3-propoxycyclophosphoranyl)-5-methyl-1-pyrroline *N*-oxide (CYPMPO) was used because CYPMPO has better trapping ability for O_2_^−•^ and *tert*-BuOO^•^ than DMPO. CYPMPO was obtained from Shidai Systems (Saitama, Japan). For the detection of singlet oxygen (^1^O_2_), 4-hydroxy-2,2,6,6-tetramethylpiperidine (4-HO-TEMP) was obtained from Tokyo Chemical Ind. The precursors and sensitizers for the formation of active species were reported elsewhere.^(8–10)^ Ginger-related antioxidant compounds (6-gingerol and zingerone (purity >98%)) were obtained from Tokyo Chemical Ind. and used as received. 6-Shogaol was prepared according to the method reported by Okamoto *et al.*^(12)^ Identification was made by ^1^H NMR (nuclear magnetic resonance) measurements (purity >97%). Acetonitrile (Wako Pure Chemical Industries Ltd., Osaka, Japan) and water purified by distillation were used as a mixture solvent.

### Methods

The essence of the MULTIS method is to photolitically generate a finite amount of free radicals (reactive oxygen species) in the absence or presence of the known amount of antioxidant-containing specimen. The decrease of active species concentration due to the sample’s antioxidant activity is quantified with ESR spin trapping method.

### Sampling procedures of ginger and ESR measurements

Yellow gingers (*Zingiber officinale*) harvested in two prefectures were used (sample A and B): the ginger harvested in October was stored at ambient temperature and 90% humidity. Liquid ginger samples (*n* = 3) were obtained through grating ginger roots followed by squeezing with a piece of cloth. The grated ginger sample in the bottle was kept at 80°C in a water-bath for 2 h. Rosemary leaves (*Majorca Pink*) were harvested in January 2017 in Okayama City, Japan. The Rosemary leaves (5 g) were chopped into small pieces, suspended in 50 ml acetonitrile, and heated at 80°C for 1 h with agitation.^(10)^ The extract was then brought to room temperature.

The ginger sample was added to the spin-trap solution that contains 3–10 mM (M = mol dm^−3^) spin trap. The amount of ginger was 0.1–0.5 vol%. The resulting solution was transferred into an ESR sample tube. Five reactive oxygen species were independently generated with UV/Vis light illumination (UV illuminator: RUVF-203S, Radical Research Inc., Hino, Tokyo, Japan). The detailed procedures for ESR measurements have been described elsewhere.^(9,10)^ Hydroxyl and alkoxyl radicals were generated with the UV illumination of H_2_O_2_ (10 mM) and AAPH (3 mM), respectively. Singlet oxygen and superoxide were formed from the photo-sensitizer pterin-6-carboxylic acid (40 µM, Wako Pure Chemical Ind.) and riboflavin (50 µM, Tokyo Chemical Ind.), respectively. Singlet oxygen was quantified by the oxidation of 4-HO-TEMP. The *tert*-BuOO^•^ radical was produced from UV-photolysis of *tert*-butylhydroperoxide (40 mM, Tokyo Chemical Ind.).^(13)^

After UV/Vis light illumination, the ESR signal intensity of active species adducts were measured. A JEOL FA200 X-band ESR spectrometer (JEOL Ltd., Akishima, Tokyo, Japan) was used to record the ESR spectra.

### Determination of antioxidant ability

The concentration of reactive oxygen species was quantified by using ESR spin trapping method. The ESR signal intensities of spin adducts with or without ginger antioxidants were measured and the active species scavenging rates were determined. To evaluate the relative scavenging rate constants of ginger-related components, the following competitive relation between spin trap and antioxidant was assumed:^(14)^

$$\text{AOx}+{\text{R}}^{\cdot }\overset{k_{\text{AOx}}}{\to }\text{Product}$$

$$\text{ST}+{\text{R}}^{\cdot }\overset{k_{\text{ST}}}{\to }\text{ST-R}$$

$$\frac{{\text{I}}_{\text{0}}-\text{I}}{\text{I}}=\frac{k_{\text{AOx}}{\left[\text{AOx}\right]}_{0}}{k_{\text{ST}}{\left[\text{ST}\right]}_{0}}$$ [1]

where I and I_0_ denote the ESR signal heights in the presence and absence of antioxidants, respectively. ESR signal intensity was assumed to be proportional to the active species concentration. The value of (I_0_ – I)/I was plotted against [AOx]_0_/[ST]_0_. A straight relationship passing through the origin was obtained for ginger antioxidant components, justifying the above competitive mechanism.

The scavenging ability of grated ginger is certainly due to the activity by multiple antioxidants. Such antioxidant components (AOx(1), AOx(2), ..., AOx(*n*)) in the ginger sample and spin trap (ST) undergo competitive scavenging reactions against free radical (R^•^) as follows:

$$\text{ST}+{\text{R}}^{\cdot }\overset{k_{\text{ST}}}{\to }\text{Spin adduct}$$

$$\text{AOx(1)}+{\text{R}}^{\cdot }\overset{k_{\text{1}}}{\to }\text{Product-1}$$

$$\begin{array}{l} \text{AOx(2)}+{\text{R}}^{\cdot }\overset{k_{\text{2}}}{\to }\text{Product-2}\\ \;\;\;\;\;\;\vdots \\ \text{AOx(}n\text{)}+{\text{R}}^{\cdot }\overset{k_{n}}{\to }\text{Product-}n \end{array}$$

Therefore, ginger’s total scavenging ability can be expressed as the sum of each component’s scavenging rate:^(9)^

$$\begin{array}{c} \frac{\nu_{\text{ginger}}}{\nu_{\text{ST}}}=\frac{{\text{I}}_{\text{0}}-\text{I}}{\text{I}}=\frac{\sum_{i}^{n}k_{i}[\text{AOx(}i\text{)}][{\text{R}}^{\cdot }]}{k_{\text{ST}}[\text{ST}][{\text{R}}^{\cdot }]}=\frac{\sum_{i}^{n}k_{i}\alpha_{i}{\left[\text{AOx}\%\right]}_{0}}{k_{\text{ST}}{\left[\text{ST(1 M)}\right]}_{0}{[\text{ST(mol)]}}_{0}}\\ =\text{β}\frac{{\text{[AOx}\%]}_{0}}{{\text{[ST(mol)]}}_{0}} \end{array}$$ [2]

where the [ ]_0_ symbol denotes the initial concentration (M) of spin traps and the ginger antioxidant components. [AOx%] and [ST (mol)] express the concentration in volume (%) and ST amount by mole, respectively. α_i_ (= [AOx(*i*)]_0_/100) and β (= *v*_ginger(1%)_/*v*_ST(1__ __M)_) are constants. Plotting (I_0_ − I)/I against [AOx%]_0_/[ST (mol)]_0_ enables us to calculate relative scavenging rate of grated ginger (100%) in 1 mM ST solution (*v*_ginger(100%)_/*v*_ST(1__ __mM)_).

## Results

### Antioxidant activities of grated ginger and components

Using the ESR peak heights I_0_ and I in the absence and presence of grated ginger, the relative scavenging rates (*v*_ginger_/*v*_ST_) against 1 mM ST were calculated using Eq. (2). The results for the ginger samples at 25°C and heated at 80°C for 2 h are listed in Table 1, together with the active species scavenging rates of rosemary extracts. The ESR measurements of antioxidant abilities were repeated 5 times for the same ginger and rosemary samples, and the error was shown as probable error. Trolox was selected as the standard scavenger and relative scavenging rates of grated ginger and rosemary extract against 100 mM trolox solution were expressed in trolox equivalent units (TEU100) (Table 1).

The relative scavenging rates for ginger samples (1 and 2) against reactive oxygen species can be expressed by using Eq. (2) as follows:

$$\frac{\nu_{\text{ginger 2}}}{\nu_{\text{ginger 1}}}=\frac{{\text{(I}}_{\text{0}}{\text{/I}}_{\text{ginger 2}}\text{)}-1}{{\text{(I}}_{\text{0}}{\text{/I}}_{\text{ginger 1}}\text{)}-1}$$ [3]

**Fig. 1:**
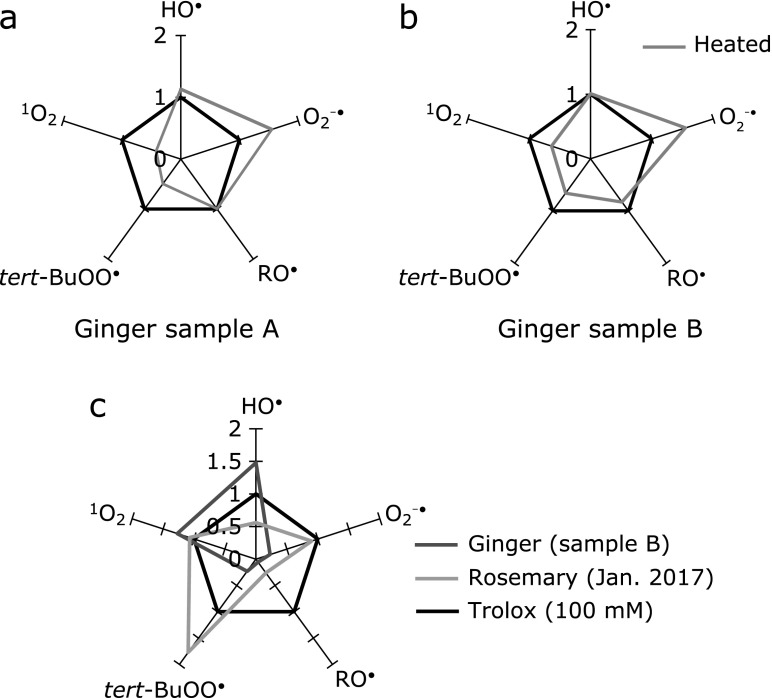
Radar chart illustration of relative scavenging rates for ginger sample A (a) and sample B (b). Radar charts show the MULTIS values in liquid ginger samples at 25°C (black line) and ones heated at 80°C (gray line). The MULTIS values at 25°C are set to 1.0, thus its radar chart is a normal pentagon (black line). (c) The relative scavenging rates of grated ginger sample B and rosemary extract harvested in Okayama region (January 2017). TEU100 (equivalent to trolox using 100 mM trolox solution as standard) was employed as a standard MULTIS number.

Fig. 1a and b show the radar chart for the scavenging rates in heat-treated ginger samples (A and B). Furthermore, Fig. 1c illustrates the radar chart for the antioxidant activities (scavenging rates) of grated ginger and rosemary extract as relative numbers with respect to the standard antioxidant trolox.

So far, the comparison of antioxidant capacity in antioxidant mixtures such as plant extracts has been hampered by the lack of standard. In the case of pure antioxidant, trolox has been used as a standard antioxidant and antioxidant capacity is often expressed in trolox equivalent unit. In this study, we used scavenging rate profile of 100 mM trolox solution (TEU100) as a standard scavenging rate profile to make radar charts.

**Fig. 2:**
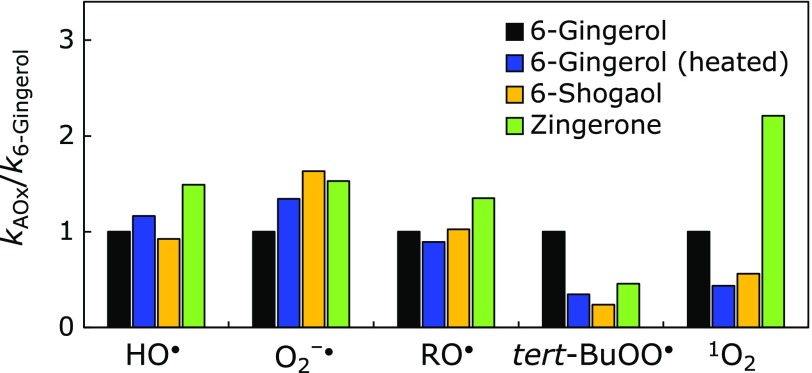
Bar graph that represents relative scavenging rate constants (*k*_AOx_/*k*_6-gingerol_) of ginger-related compounds (6-gingerol, 6-shogaol, and zingerone) against 5 reactive oxygen species. Those numbers are listed in Table 2.

Because gingerol, shogaol, and zingerone have been shown to be antioxidant components in ginger,^(3,5)^ we determined the relative scavenging rate constant (*k*_AOx_/*k*_ST_) of these pure compounds. The activity is presented in trolox equivalent units (TEU (*k*_AOx_/*k*_trolox_)) (Table 2). Further, Fig. 2 shows the relative rate constants of 3 ginger-related compounds against 6-gingerol.

## Discussion

Using the ESR spin-trapping based MULTIS method, we have determined the scavenging abilities of grated ginger against five reactive oxygen species. By heat treatment, we showed that grated ginger increased O_2_^−•^ scavenging ability and decreased the ^1^O_2_ and *tert*-BuOO^•^ scavenging abilities. Furthermore, scavenging activities of ginger-related antioxidant compounds (6-gingerol, 6-shogaol, and zingerone) were evaluated against five reactive oxygen species.

It is noted that upon heating the sample at 80°C for 2 h, there was about 1.5-fold increase for superoxide scavenging in all ginger samples (Fig. 1a and b). Conversely, the antioxidant activities for singlet oxygen and peroxyl radical decreased. The pungent compounds in ginger, such as shogaol and zingerone, have been shown to be chemically produced from gingerol during thermal processing and long-term storage.^(15,16)^ Also, Jolad *et al.*^(5)^ demonstrated that there was thermal degradation of gingerol into zingerone and shogaol, that is the dehydrated form of gingerol. In the separate experiments, the MULTIS antioxidant activities of pure 6-gingerol, 6-shogaol, and zingerone were determined and the results are listed in Table 2.

Fig. 2 is a bar graph illustration of the numbers in Table 2, indicating that: (1) The antioxidant abilities of 6-shogaol for singlet oxygen and peroxyl radical are markedly low compared with those of 6-gingerol; (2) Although zingerone’s scavenging ability is modest, it possesses the highest singlet oxygen scavenging ability among the test ginger components. (3) It is noteworthy that the heat treatment (80°C, 2 h) lowered 6-gingerol’s scavenging ability against singlet oxygen and peroxyl radical. Similar tendency was found in 6-shogaol activity. The above results are in line with the fact that 6-gingerol is transformed into 6-shogaol after heating. We concluded that the heat-mediated change in the scavenging ability in grated ginger is caused by the transformation of the main component 6-gingerol into 6-shogaol.

The leaves of rosemary (*Rosmarinus officinalis*) have been recognized as having plant compositions that possess high antioxidant activities.^(10)^ Our previous study established a method to express MULTIS values for the hydrophilic antioxidants in rosemary leaves.^(10)^ Using trolox as a standard antioxidant, the MULTIS study makes it possible to make quantitative comparison between various foods with antioxidant activity. Thus, we compared MULTIS profiles of rosemary leaves and ginger roots (total of hydrophilic and lipophilic antioxidants). We set MULTIS profile of 100 mM trolox solution as a standard and made radar charts for ginger and rosemary in one illustration (Fig. 1c). This chart clearly indicates that ginger and rosemary each has specific scavenging pattern. Rosemary is good at scavenging singlet oxygen and peroxyl radical, while ginger is good at scavenging hydroxyl radical and singlet oxygen.

The existing data for antioxidants have made it possible to speculate the scavenging mechanism by ginger’s antioxidant components. 6-Gingerol showed relatively high scavenging ability against hydroxyl and singlet oxygen (Table 2). Previously, HO^•^ scavenging by 6-gingerol was shown to be taken place predominantly via radical adduct formation (RAF) mechanism.^(17)^ Also, previous studies showed that ^1^O_2_ quenching reaction by flavonoid antioxidants proceeds via a charge-transfer intermediate.^(18)^ Because antioxidants having smaller oxidation potential *E*ox tends to show higher ^1^O_2_ scavenging rates, the relatively small *E*ox of 6-gingerol (0.39 V)^(19)^ suggests that 6-gingerol is an effective quencher for ^1^O_2_ and that 6-gingerol’s ^1^O_2_ quenching reaction proceeds via charge-transfer mechanism.

**Fig. 3:**
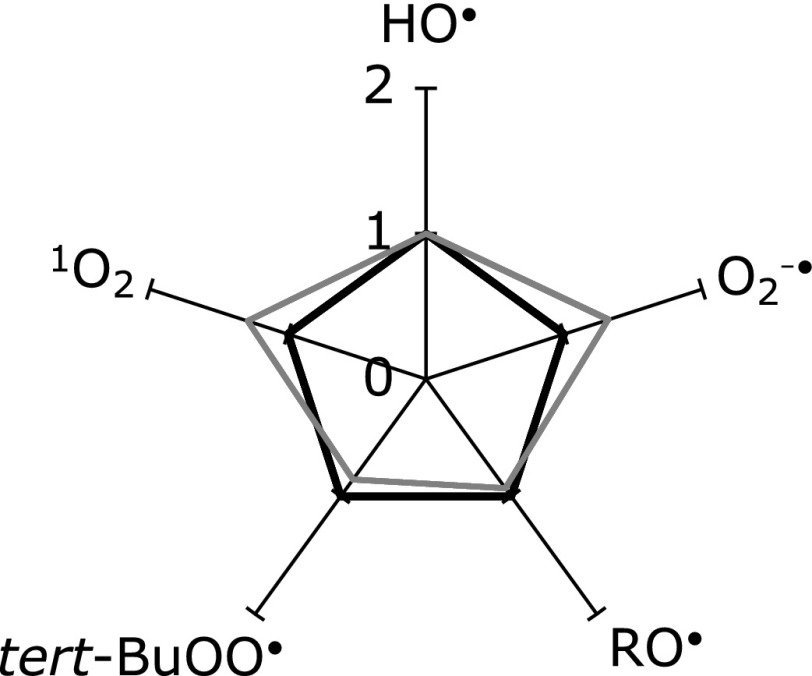
Radar chart illustration of relative scavenging rates for the MULTIS values estimated from the component quantity of 6-gingerol + 6-shogaol (gray line) against grated ginger sample A (black line). The HO^•^ scavenging ability of 6-gingerol + 6-shogaol is adjusted to 1.0.

Finally, Fig. 3 shows a radar chart illustration for the antioxidant abilities of the present ginger sample A, together with the MULTIS values estimated from the component quantity of 6-gingerol (29.4% (w/w)) and 6-shogaol (4.3% (w/w)).^(3)^ The HO^•^ scavenging ability of 6-gingerol + 6-shogaol is adjusted to 1.0, because it has been shown that scavenging abilities of antioxidant compounds against HO^•^ radical was unselective.^(14)^ The scavenging ability of 6-gingerol is nearly the same as that of the grated ginger sample, suggesting that 6-gingerol plays the major role in the overall scavenging activity.

## Conclusion

The present study demonstrated that the heat treatments of ginger specimens either increase or decrease the scavenging activity, depending on the kind of reactive oxygen species subject to scavenging. This means that unlike other antioxidant-containing vegetables, ginger’s antioxidant activity is not necessarily lost by cooking at elevated temperatures. Active species acting as antioxidant capacity of ginger was revealed. In addition, we show that the illustration of radar charts of different plant extracts such as ginger and rosemary provides insightful information on specific scavenging capacity.

## Figures and Tables

**Table 1:** Relative scavenging abilities of grated ginger against five reactive oxygen species in acetonitrile and water (1:1, v/v) mixture at room temperature

Ginger	X = *v*_ginger (100%)_/*v*_ST (1 mM)_^a)^ (TEU100 values in parenthesis)				
	HO^•^^b)^	O_2_^−•^	RO^•^^c)^	*tert*-BuOO^•^^d)^	^1^O_2_
Sample A	3,360 ± 180	1,400 ± 102	345 ± 18	655 ± 31	233,000 ± 9,000
	(1.05)	(0.17)	(0.03)	(0.23)	(0.99)
Sample A (heated)	3,810 ± 160	2,180 ± 78	344 ± 16	324 ± 10	98,000 ± 3,900
	(1.19)	(0.26)	(0.03)	(0.11)	(0.42)
Sample B	4,720 ± 110	1,930 ± 98	645 ± 32	662 ± 29	299,000 ± 12,000
	(1.48)	(0.23)	(0.06)	(0.23)	(1.27)
Sample B (heated)	4,800 ± 120	2,980 ± 120	535 ± 10	440 ± 9	190,000 ± 11,000
	(1.50)	(0.36)	(0.05)	(0.16)	(0.81)
Rosemary^e)^ (Jan. 2017)	1,780 ± 170	7,570 ± 830	3,010 ± 50	5,000 ± 250	251,500 ± 18,400
	(0.56)	(0.90)	(0.26)	(1.77)	(1.07)
Trolox (100 mM)	3,190 ± 330	8,390 ± 230	11,500 ± 300	2,830 ± 130	236,000 ± 7,000
	(1)	(1)	(1)	(1)	(1)

^a)^ST = DMPO for HO^•^ and RO^•^, CYPMPO for O_2_^−•^ and *tert*-BuOO^•^, and 4-HO-TEMP for ^1^O_2_. ^b)^The HO^•^ scavenging ability in an acetonitrile-water mixture (3:7, v/v). ^c)^Alkoxyl radical derived from AAPH. ^d)^Peroxyl radical derived from *tert*-butylhydroperoxide. ^e)^Rosemary harvested in January 2017.

**Table 2:** Relative scavenging rate constants *k*_AOx_/*k*_ST_ and TEU values of ginger’s antioxidant components in a mixture of acetonitrile and water (1:1, v/v) at room temperature

Antioxidant	*k*_AOx_/*k*_ST_, TEU values in the parenthesis				
	HO^•^^a)^ vs DMPO	O_2_^−•^ vs CYPMPO	RO^•^ vs DMPO	*tert*-BuOO^•^ vs CYPMPO	^1^O_2_ vs 4-OH-TEMP
6-Gingerol	45.0 ± 2.3	22.8 ± 1.2	4.24 ± 0.14	8.23 ± 0.24	4,230 ± 130
	(1.41)	(0.272)	(0.037)	(0.291)	(1.79)
6-Gingerol (heated)	52.4 ± 1.9	30.6 ± 1.5	3.79 ± 0.18	2.84 ± 0.13	1,840 ± 90
	(1.64)	(0.365)	(0.033)	(0.100)	(0.780)
6-Shogaol	41.6 ± 2.9	37.2 ± 2.0	4.34 ± 0.13	1.96 ± 0.09	2,380 ± 180
	(1.30)	(0.443)	(0.038)	(0.069)	(1.01)
Zingerone	67.1 ± 2.3	34.9 ± 1.8	5.73 ± 0.22	3.75 ± 0.19	9,350 ± 410
	(2.10)	(0.416)	(0.050)	(0.133)	(3.96)
Trolox	31.9 ± 3.3	83.9 ± 2.3	115 ± 3	28.3 ± 1.3	2,360 ± 70
	(1)	(1)	(1)	(1)	(1)

^a)^The HO^•^ scavenging ability in an acetonitrile-water mixture (3:7, v/v).

## References

[1] Yang G, Zhong L, Jiang L (2011). 6-Gingerol prevents patulin-induced genotoxicity in HepG2 cells. Phytother Res.

[2] Wei QY, Ma JP, Cai YJ, Yang L, Liu ZL (2005). Cytotoxic and apoptotic activities of diarylheptanoids and gingerol-related compounds from the rhizome of Chinese ginger. J Ethnoparmacol.

[3] Si W, Chen YP, Zhang J, Chen ZY, Chung HY (2018). Antioxidant activities of ginger extract and its constituents toward lipids. Food Chem.

[4] Aeschbach R, Löliger J, Scott BC (1994). Antioxidant actions of thymol, carvacrol, 6-gingerol, zingerone and hydroxytyrosol. Food Chem Toxicol.

[5] Jolad SD, Lantz RC, Solyom AM, Chen GJ, Bates RB, Timmermann BN (2004). Fresh organically grown ginger (*Zingiber officinale*): composition and effects on LPS-induced PGE_2_ production. Phytochemistry.

[6] Dugasani S, Pichika MR, Nadarajah VD, Balijepalli MK, Tandra S, Korlakunta JN (2010). Comparative antioxidant and anti-inflammatory effects of [6]-gingerol, [8]-gingerol, [10]-gingerol and [6]-shogaol. J Ethnopharmacol.

[7] Ishikawa H, Matsumoto K, Ukeda H, Shimamura T, Matsufuji H, Yamazaki T (2010). Methods for the estimation of antioxidant capacities in foods. Food Food Ingredients J Jpn.

[8] Oowada S, Endo N, Kameya H, Shimmei M, Kotake Y (2012). Multiple free-radical scavenging capacity in serum. J Clin Biochem Nutr.

[9] Sueishi Y, Kamogawa E, Kimura A (2017). Multiple free-radical scavenging (MULTIS) capacity in cattle serum. J Clin Biochem Nutr.

[10] Sueishi Y, Sue M, Masamoto H (2018). Seasonal variation of oxygen radical scavenging ability in rosemary leaf extract. Food Chem.

[11] Kanno T, Kawamura S, Harada E, Kameya H, Ukai M, Osawa T (2013). Radical scavenging ability by ESR spin trapping and oxygen radical absorbance capacity of hot water extracts from mushroom. Nippon Shokuhin Kogaku Kogaku Kaishi.

[12] Okamoto M, Irii H, Tahara Y (2011). Synthesis of a new [6]-gingerol analogue and its protective effect with respect to the development of metabolic syndrome in mice fed a high-fat diet. J Med Chem.

[13] Bors W, Michel C, Stettmaier K (1992). Radical species produced from the photolytic and pulse-radiolytic degradation of *tert*-butyl hydroperoxide. An EPR spin trapping investigation. J Chem Soc Perkin Trans 2.

[14] Sueishi Y, Hori M, Ishikawa M (2014). Scavenging rate constants of hydrophilic antioxidants against multiple reactive oxygen species. J Clin Biochem Nutr.

[15] Connell DW, Sutherland MD (1969). A re-examination of gingerol, shogaol, and zingerone, the pungent principles of ginger (*Zigiber officinale* Roscoe). Aust J Chem.

[16] Bahttarai S, Tran VH, Duke CC (2001). The stabilities of gingerol and shogaol in aqueous solutions. J Pharm Sci.

[17] Iuga C, Alvarez-Idaboy JR, Russo N (2012). Antioxidant activity of trans-resveratrol toward hydroxyl and hydroperoxyl radicals: a quantum chemical and computational kinetics study. J Org Chem.

[18] Mukai K, Nagai S, Ohara K (2005). Kinetic study of the quenching reaction of singlet oxygen by tea catechins in ethanol solution. Free Rad Biol Med.

[19] Liu C, Tang D, Gao Y (2007). Research on electrochemistry behaviors of gingerol. Shipin Kexue (Beijing, China).

